# Red fluorescent genetically encoded Ca^2+^ indicators for use in mitochondria and endoplasmic reticulum

**DOI:** 10.1042/BJ20140931

**Published:** 2014-10-23

**Authors:** Jiahui Wu, David L. Prole, Yi Shen, Zhihong Lin, Aswini Gnanasekaran, Yingjie Liu, Lidong Chen, Hang Zhou, S. R. Wayne Chen, Yuriy M. Usachev, Colin W. Taylor, Robert E. Campbell

**Affiliations:** *Department of Chemistry, University of Alberta, Edmonton, Alberta, Canada, T6G 2G2; †Department of Pharmacology, University of Cambridge, Cambridge CB2 1PD, U.K.; ‡Department of Pharmacology, University of Iowa Carver College of Medicine, Iowa City, IA 52242, U.S.A.; §Department of Physiology and Pharmacology, Libin Cardiovascular Institute of Alberta, University of Calgary, Calgary, Alberta, Canada, T2N 4N1; ¶Department of Biochemistry and Molecular Biology, Libin Cardiovascular Institute of Alberta, University of Calgary, Calgary, Alberta, Canada, T2N 4N1

**Keywords:** endoplasmic reticulum (ER), fluorescence Ca^2+^ imaging, GCaMP, mitochondrion, multicolour imaging, red fluorescent genetically encoded Ca^2+^ indicator for optical imaging (R-GECO), [Ca^2+^]_i_ and [Ca^2+^]_mt_, free Ca^2+^ concentration in cytosol and mitochondrial matrix, respectively, CaM, calmodulin, cpFP, circularly permuted fluorescent protein, DMEM, Dulbecco’s modified Eagle’s medium, DRG, dorsal root ganglion, ER, endoplasmic reticulum, FP, fluorescent protein, FRET, Förster resonance energy transfer, GFP, green fluorescent protein, HBS, Hepes-buffered saline, HEK, human embryonic kidney, LAR-GECO, low-affinity red fluorescent genetically encoded Ca^2+^ indicator for optical imaging, LED, light-emitting diode, NA, numerical aperture, NTA, nitrilotriacetic acid, RFP, red fluorescent protein, RyR2, type 2 ryanodine receptor, SERCA, sarcoplasmic/endoplasmic reticulum Ca^2+^-ATPase, SOICR, store overload-induced Ca^2+^ release, SR, sarcoplasmic reticulum

## Abstract

Ca^2+^ is a key intermediary in a variety of signalling pathways and undergoes dynamic changes in its cytoplasmic concentration due to release from stores within the endoplasmic reticulum (ER) and influx from the extracellular environment. In addition to regulating cytoplasmic Ca^2+^ signals, these responses also affect the concentration of Ca^2+^ within the ER and mitochondria. Single fluorescent protein-based Ca^2+^ indicators, such as the GCaMP series based on GFP, are powerful tools for imaging changes in the concentration of Ca^2+^ associated with intracellular signalling pathways. Most GCaMP-type indicators have dissociation constants (*K*_d_) for Ca^2+^ in the high nanomolar to low micromolar range and are therefore optimal for measuring cytoplasmic [Ca^2+^], but poorly suited for use in mitochondria and ER where [Ca^2+^] can reach concentrations of several hundred micromolar. We now report GCaMP-type low-affinity red fluorescent genetically encoded Ca^2+^ indicators for optical imaging (LAR-GECO), engineered to have *K*_d_ values of 24 μM (LAR-GECO1) and 12 μM (LAR-GECO1.2). We demonstrate that these indicators can be used to image mitochondrial and ER Ca^2+^ dynamics in several cell types. In addition, we perform two-colour imaging of intracellular Ca^2+^ dynamics in cells expressing both cytoplasmic GCaMP and ER-targeted LAR-GECO1. The development of these low-affinity intensiometric red fluorescent Ca^2+^ indicators enables monitoring of ER and mitochondrial Ca^2+^ in combination with GFP-based reporters.

## Abstract 2

This paper describes the engineering of two red fluorescent genetically encoded Ca^2+^ indicators with low affinities. These indicators enable monitoring of mitochondrial and ER Ca^2+^ dynamics in combination with a green fluorescent indicator.

## INTRODUCTION

As a ubiquitous intracellular messenger, Ca^2+^ has essential physiological roles in a variety of cellular processes including fertilization and development, muscle contraction and neurotransmitter release [1]. The development and subsequent application of genetically encoded Ca^2+^ indicators based on fluorescent proteins (FPs) has revolutionized the study of intracellular Ca^2+^ dynamics. There are two predominant classes of FP- based Ca^2+^ indicator: the cameleon-type based on Förster resonance energy transfer (FRET) [2]; and the GCaMP-type based on a single circularly permuted FP (cpFP) [3,4]. Cameleon-type Ca^2+^ indicators are composed of a genetic fusion of calmodulin (CaM) and a short Ca^2+^-CaM-binding peptide (e.g. M13), flanked by a blue-shifted donor FP and a red-shifted acceptor FP. As FRET is strongly dependent on the distance between donor and acceptor fluorophores, the conformational change associated with the Ca^2+^-dependent interaction of CaM and M13 leads to a change in FRET efficiency and a ratiometric change in the fluorescence signal [2]. In contrast, GCaMP-type indicators consist of a cpFP genetically fused to an N-terminal M13 peptide and a C-terminal CaM domain [3,4]. The Ca^2+^-dependent interaction of CaM and M13 modifies the environment of the FP chromophore such that the fluorescence undergoes an intensiometric increase.

The cameleon and GCaMP classes of indicators are each associated with specific advantages and disadvantages. For example, GCaMP-type indicators have larger signal changes and a wider range of fluorescence hues [5–9], whereas cameleons tend to have smaller signal changes at each wavelength, but benefit from inherently ratiometric signal changes. Of particular relevance to the present study, cameleons have traditionally also been more amenable to genetic ‘tuning’ of the equilibrium dissociation constants for Ca^2+^ (*K*_d_) to provide variants optimized for use in environments containing low [10] or high [11–13] concentrations of Ca^2+^. Accordingly, certain cameleon-type Ca^2+^ indicators have been useful for imaging Ca^2+^ dynamics within the endoplasmic reticulum (ER) and sarcoplasmic reticulum (SR), where concentrations of Ca^2+^ are relatively high [2,11,14]. These organelles are intracellular Ca^2+^ reservoirs in eukaryotic cells and therefore play central roles in Ca^2+^ signalling [1,15,16].

Although a relatively large number of improved cameleon- and GCaMP-type indicators have been reported, only a small subset has been optimized for use in environments containing high concentrations of Ca^2+^, such as the ER and SR [15,17]. Likewise, there are relatively few variants optimized for use in mitochondria, where concentrations of Ca^2+^ can change over three orders of magnitude (~0.1 μM to ~100 μM) [12,18,19]. The primary reason for the lack of appropriate GCaMP-type indicators is that, due to their more intricate sensing mechanism, mutations in the CaM domain that could potentially increase the *K*_d_ of the indicator are also likely to interfere with the mechanism by which fluorescence is modulated. Indeed, our previous efforts to decrease the Ca^2+^ affinity of GCaMP-type indicators were hampered by seemingly unavoidable and dramatic decreases in the performance of the indicator.

The two most useful cameleon-type indicators engineered to have decreased Ca^2+^ affinity are D1ER [11] and D4cpv [12]. The Ca^2+^-sensing domains of both D1ER and D4cpv were engineered by redesigning the interaction between CaM and M13 such that they would interact with each other, but not with endogenous binding partners. Although the redesign successfully produced an orthogonal CaM–M13 pair, it also decreased the apparent affinity for Ca^2+^ to ~60 μM for both indicators. However, as these FRET-based Ca^2+^ indicators contain CFP and YFP, it is generally impractical for combined use with GFP variants in multicolour imaging. More recently, a green Ca^2+^ indicator named CatchER, based on a single GFP, was reported [20]. In this design, three β-strands in GFP were engineered to bind Ca^2+^ directly, causing a change in fluorescence. However, the fluorescence change of CatchER upon binding to Ca^2+^ is relatively small (less than 2-fold), which limits its utility [20]. A Ca^2+^ sensor termed GAP1, based on a GFP–aequorin fusion protein, has also been reported to exhibit an excitation ratiometric response to changes in the concentrations of Ca^2+^ within ER and mitochondria [21]. A shared drawback of the cameleons, CatchER and GAP1 is that they require use of blue light for excitation, which leads to relatively high levels of autofluorescence and phototoxicity, and prohibits simultaneous imaging with green fluorescent indicators. Longer wavelength indicators based on a RFP, rather than a GFP, are associated with lower background fluorescence, lower phototoxicity and deeper tissue penetration. Accordingly, there remains a substantial need for RFP-based Ca^2+^ indicators optimized for imaging of Ca^2+^ dynamics in organelles containing relatively high concentrations of Ca^2+^, such as ER, SR and mitochondria.

In the present paper, we describe the combined use of rational design and directed evolution for the engineering of two RFP-based Ca^2+^ indicators for imaging of Ca^2+^ dynamics within mitochondria and ER. These new indicators show high sensitivity to changes in the concentration of Ca^2+^ within organelles that contain relatively high concentrations of Ca^2+^, and are useful for multicolour imaging when paired with green fluorescent indicators.

## EXPERIMENTAL

### Engineering of LAR-GECO1 and LAR-GECO1.2

R-GECO1 and R-GECO1.2 in pTorPE [5] were used as the initial templates for engineering low-affinity red fluorescent genetically encoded Ca^2+^ indicators for optical imaging (LAR-GECOs). Point mutations and randomizations of specific amino acids were introduced using a QuikChange® Lightning Site-Directed Mutagenesis Kit (Agilent) as per the manufacturer's instructions. Oligonucleotides containing specific mutations were designed using an online mutagenesis primer design program (Agilent). Random mutagenesis was performed by error-prone PCR amplification [22]. To construct mammalian expression plasmids, LAR-GECOs in the pBAD/His B vector (Life Technologies) were used as the templates. The ER-targeted GECO constructs were generated using primers containing an ER-targeting sequence (MLLPVLLLGLLGAAAD) [23] and an ER-retention signal sequence (KDEL) [24]. The PCR products were subjected to digestion with BamHI and EcoRI restriction enzymes (Thermo Scientific). The digested DNA fragments were ligated with a modified pcDNA3 plasmid that had previously been digested with the same two enzymes. For mito-LAR-GECO1.2, two copies of the targeting sequence (MSVLTPLLLRGLTGSARRLPVPRAKIHSLGDP) from cytochrome *c* oxidase VIII were added to the N-terminal end of LAR-GECO1.2. Plasmids were purified with the GeneJET miniprep kit (Thermo Scientific) and then sequenced using the BigDye Terminator Cycle Sequencing kit (Applied Biosystems).

A custom imaging system [25] was used for screening LAR-GECO variants in the context of *Escherichia coli* colonies. Specifically, LAR-GECO variants in the pBAD/His B vector (Life Technologies) were electroporated into the *E. coli* strain DH10B (Invitrogen). *E. coli* containing these variants were then cultured on 10-cm LB agar Petri dishes supplemented with 400 μg/ml ampicillin (Sigma) and 0.02% L-arabinose (Alfa Aesar) at 37°C overnight. These Petri dishes were then placed at room temperature for 24 h before screening. During screening, an image was captured for each Petri dish by using an excitation filter of 542/27 nm to illuminate *E. coli* colonies expressing LAR-GECO variants and an emission filter of 609/57 nm. The colonies with the highest fluorescence intensity (top 0.1%) in each image were then picked and cultured in 4 ml liquid LB with 100 μg/ml ampicillin and 0.02% L-arabinose at 37°C overnight. Proteins were then extracted from the liquid LB culture and subjected to a secondary screen using a Safire2 fluorescence microplate reader (Tecan).

### *In vitro* characterization of LAR-GECO1 and LAR-GECO1.2

For detailed characterization of LAR-GECOs, proteins were expressed and purified as previously described [6]. Spectral measurements were performed in solutions containing 10 mM EGTA or 10 mM Ca^2+^/NTA (nitrilotriacetic acid), 30 mM Mops and 100 mM KCl, pH 7.2. For determination of fluorescence quantum yield (ϕ), mCherry [26] was used as a standard. Procedures for measurement of fluorescence quantum yield, molar absorption coefficient (ε), p*K*_a_, *K*_d_ for Ca^2+^, and Ca^2+^-association kinetics have been described previously [6]. For Ca^2+^ titration, Ca^2+^/EDTA, Ca^2+^/HEDTA [*N*-(2-hydroxyethyl)ethylenediamine-*N*,*N*′,*N*′-triacetic acid], and Ca^2+^/NTA buffers were prepared by mixing Ca^2+^-saturated and Ca^2+^-free buffers (30 mM Mops, 100 mM KCl and 10 mM chelating reagent, pH 7.2, either with or without 10 mM Ca^2+^) to achieve buffer-free Ca^2+^ concentrations from 0 to 1.13 mM.

### Imaging ER luminal Ca^2+^ with ER-LAR-GECO1, CatchER and ER-GAP1

HeLa, human embryonic kidney (HEK)-293 and U2-OS cells were cultured in Dulbecco's modified Eagle's medium (DMEM)/F12 medium (Life Technologies) supplemented with FBS (10%, Sigma–Aldrich) at 37°C in air containing 5% CO_2_. Cells were seeded on fibronectin-coated, glass-bottomed culture dishes and transiently transfected after 24 h with plasmids using Transit-LT1 (Mirus Bio). Cells were imaged 24–48 h after transfection, using an Olympus IX83 microscope with a ×100/1.49 numerical apesture (NA) objective, at 20°C in Hepes-buffered saline (HBS). HBS had the following composition: 132 mM NaCl, 5 mM KCl, 2 mM MgCl_2_, 10 mM D-glucose, 10 mM Hepes and 2 mM CaCl_2_, pH 7.2. Ca^2+^ was omitted from nominally Ca^2+^-free HBS. Cells were illuminated alternately, via a 405/488/561/647-nm quad band dichroic/emitter (TRF89902, Chroma Technology), with a 559-nm light-emitting diode (LED) (Spectra X, Lumencor) for visualizing ER-LAR-GECO1 and a 488-nm diode laser (Coherent) for visualizing ER-GFP and CatchER. Ratiometric measurements of ER-GAP1 fluorescence were achieved by illuminating cells alternately with a 395-nm LED (Spectra X, Lumencor) or a 488-nm laser and measuring fluorescence emission at 520–550 nm using a 535/30 nm emission filter (Chroma Technology). With the filters used there was no significant cross-talk between blue, green and red channels. Images were acquired with an iXon Ultra electron multiplying charge-coupled device (EMCCD) camera (512×512 pixels) (Andor Technology) and images were processed using MetaMorph (Molecular Devices). All records were corrected for background fluorescence determined from regions outside cells. Fluorescence changes from regions of interest were expressed as *F*/*F*_0_, where *F*_0_ and *F* denote the average fluorescence at the start of the experiment (*F*_0_) and at each time point (*F*), respectively. Ratios of the ER-GAP1 fluorescence with alternating excitation at 488 nm and 395 nm were expressed as *R* (fluorescence intensity with 488-nm excitation/fluorescence intensity with 395-nm excitation), and *R*_0_ was defined as the average value of *R* at the start of the experiment.

### Imaging store overload-induced Ca^2+^ release in HEK-293 cells using ER-LAR-GECO1 and D1ER

HEK-293 cells stably expressing wild-type murine type 2 ryanodine receptors (RyR2) (GenBank® accession number: AF295105) were grown to 95% confluence in a 75-cm^2^ flask, split with PBS, and plated in 100-mm tissue culture dishes at ~10% confluence for 18–20 h before transfection with cDNA encoding D1ER or ER-LAR-GECO1 using the calcium phosphate precipitation method. After 24 h, the growth medium was changed to an induction medium containing 1 μg/ml tetracycline (Sigma). After induction for ~22 h, the cells were perfused continuously with KRH buffer (125 mM NaCl, 5 mM KCl, 1.2 mM KH_2_PO_4_, 6 mM glucose, 1.2 mM MgCl_2_ and 25 mM Hepes, pH 7.4) containing various concentrations of CaCl_2_ (0 mM, 1 mM and 2 mM) and tetracaine (1 mM) for estimating the store capacity or caffeine (20 mM) for estimating the minimum store level by depleting the ER Ca^2+^ stores at room temperature (23°C). For D1ER recordings, images were captured with Compix Simple PCI 6 software every 2 s using an inverted microscope (Nikon TE2000-S) equipped with an S-Fluor ×20/0.75 NA objective. The filters used for D1ER imaging were excitation, 436±20 nm for CFP, and 500±20 nm for YFP; and emission, 465±30 nm for CFP, and 535±30 nm for YFP, with a dichroic mirror (500 nm). FRET was determined from the ratio of the light emission at 535 nm and 465 nm. For ER-LAR-GECO1 recordings, images were captured using a Nikon A1R confocal system. The cells were excited using a diode laser (561 nm) and fluorescence emission was detected at 570–620 nm.

### Imaging LAR-GECO1.2 in mitochondria of neurons

Primary dorsal root ganglion (DRG) neuron cultures were prepared from adult C57BL/6J mice (8–12 weeks of age) and transfected with cDNA plasmids using an Amaxa nucleofection system (Lonza) as described previously [27,28]. Primary cultures of hippocampal neurons were prepared from neonatal (P0–P1) C57BL/6J mice and transfected with cDNA using Lipofectamine 2000 (Life Technologies) using the same protocol as previously described [27]. DRG or hippocampal neurons transfected with the mito-LAR-GECO1.2 plasmid were loaded with fura 2 acetoxymethyl ester (2 μM for 30 min) to perform simultaneous monitoring of the Ca^2+^ concentration within mitochondria ([Ca^2+^]_mt_) and in the cytosol ([Ca^2+^]_i_). Cells were then placed in a chamber for flowthrough perfusion and mounted on an inverted IX-71 microscope (Olympus). Cells were perfused with Hepes-buffered Hanks balanced salt solution (HBSS) composed of 140 mM NaCl, 5 mM KCl, 1.3 mM CaCl_2_, 0.4 mM MgSO_4_, 0.5 mM MgCl_2_, 0.4 mM KH_2_PO_4_, 0.6 mM NaHPO_4_, 3 mM NaHCO_3_, 10 mM glucose and 10 mM Hepes, pH 7.4 with NaOH (310 mOsm/kg with sucrose). Fluorescence was excited sequentially at 340 nm (12 nm bandpass) 380 nm (12 nm bandpass), and 550 nm (12 nm bandpass) using a Polychrome V monochromator (TILL Photonics) and focused on the cells via a ×40/1.35 NA oil-immersion objective (Olympus). Fluorescence emission was separated from excitation by using a dual fluorophore beamsplitter FF493/574-Di01 (Semrock) and collected with a dual band emission filter FF01-512/630 (Semrock) using an IMAGO charge-coupled device (CCD) camera (640×480 pixels; TILL Photonics). 2×2 binning was used for acquisition (1 pixel ~500 nm). A series of 340 nm, 380 nm and 550 nm images was acquired every 2 s. [Ca^2+^]_i_ was calculated from the fluorescence ratio (*R*=*F*_340_/*F*_380_) using the formula [Ca^2+^]_i_=*K*_d_*β*(*R*−*R*_min_)/(*R*_max_−*R*), where *K*_d_ is dissociation constant and *β* is the scaling factor. *R*_min_, *R*_max_ and *β* were calculated by application of 10 μM ionomycin in either Ca^2+^-free buffer (1 mM EGTA) or in HBSS (1.3 mM Ca^2+^). [Ca^2+^]_mt_ changes were quantified as Δ*F*/*F*_0_=(*F*−*F*_0_)/*F*_0_×100% where *F* is the fluorescence intensity at each time point (*λ*_ex_=550 nm) and *F*_0_ is the fluorescence intensity in the resting cell before experimental manipulations. [Ca^2+^]_i_ data were analysed using TILLvisION (TILL Photonics) software. To stimulate neurons by depolarization, the extracellular KCl concentration was elevated by replacing equimolar amounts of extracellular NaCl with KCl.

### Dual-colour imaging of cytosolic and ER luminal Ca^2+^

For dual-colour imaging of ER-LAR-GECO1 and Cyto-GCaMP3, HeLa cells (CCL2 line; A.T.C.C.) were cultured on collagen-coated 35-mm glass-bottomed dishes (Matsunami) until they reached 40–60% confluency. Transfection was performed by incubating HeLa cells with a mixture of 1 μg of plasmid DNA and 3 μl of Lipofectamine 2000 (Life Technologies) for 2 h. After incubation, the medium was changed to DMEM supplemented with FBS (10%), GlutaMAX™ (2 mM, Invitrogen), penicillin-G potassium salt (50 units/ml) and streptomycin sulfate (50 μg/ml). The cells were incubated for 24 h at 37°C in air containing 5% CO_2_. Prior to imaging, culture medium was changed to HBSS with 25 mM Hepes. Widefield imaging was performed on an inverted Nikon Eclipse Ti microscope equipped with a 200 W metal halide lamp (PRIOR Lumen), ×20/0.45 and ×60/1.40 NA objectives (Nikon), and a 16-bit QuantEM 512SC EMCCD camera (Photometrics). A filter set of 470/40 nm (excitation), 525/50 nm (emission), and 495 nm (dichroic) was used for Cyto-GCaMP3. Another filter set of 545/30 nm (excitation), 620/60 nm (emission) and 570 nm (dichroic) was used for ER-LAR-GECO1. For time-lapse imaging, HeLa cells were treated with 100 μM histamine.

For characterizing ER-LAR-GECO1 with Cyto-G-GECO1.1 in cultured human cells, cells were seeded on fibronectin-coated glass-bottomed culture dishes and transiently transfected after 24 h with plasmids using Transit-LT1 (Mirus Bio). Cells were imaged 24–48 h after transfection using an Olympus IX81 microscope with a ×60/1.45 NA objective, at 20°C in HBS or nominally Ca^2+^-free HBS. Cells were illuminated with 488 nm (for Cyto-G-GECO1.1) or 561 nm (for ER-LAR-GECO1) diode lasers (Olympus), using U-MNIBA (Olympus; excitation 470–495 nm, emission 510–550 nm) and LF561A (Semrock; excitation 550–570 nm, emission 580–630 nm) filters, respectively. With the filters used there was no significant cross-talk between green and red channels. Images were acquired with an iXon 897 EMCCD camera (Andor Technology) and processed using cellR software (Olympus). All records were corrected for background fluorescence determined from regions outside cells.

## RESULTS AND DISCUSSION

### Engineering of LAR-GECO1 and LAR-GECO1.2

**Figure 1:**
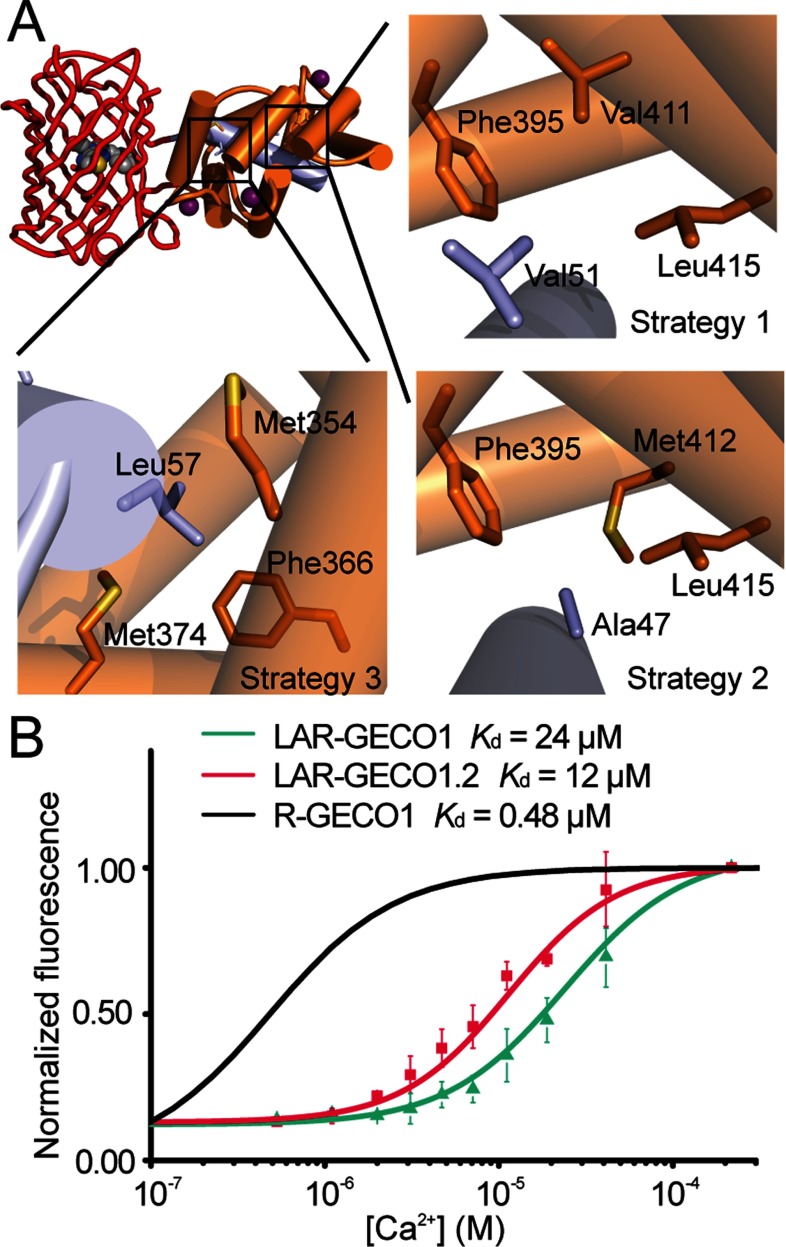
Strategies for development of LAR-GECO1 and LAR-GECO1.2 and their dissociation constants (**A**) Strategies 1, 2 and 3 for making orthogonal CaM (orange)–M13 (grey) pairs with side chains of R-GECO1 (PDB code 4I2Y) shown in stick format [8]. (**B**) Normalized fluorescence intensities of LAR-GECO1, LAR-GECO1.2 and R-GECO1 as a function of free Ca^2+^ concentrations in buffer (10 mM Mops and 100 mM KCl, pH 7.2). The black line for R-GECO1 is a curve fitted to data reported previously [5], with *K*_d_=482 nM and Hill slope=2.06.

To engineer new Ca^2+^ sensors with decreased affinity, we initially considered two single RFP-based Ca^2+^ indicators, R-GECO1 (*K*_d_=0.48 μM) [5] and R-GECO1.2 (*K*_d_=1.2 μM) [6], as potential templates. As an initial test of their ability to function in the ER, we fused each of these indicators to ER-targeting [23] and ER-retention sequences [24], and expressed them in HeLa cells. Both indicators showed ER localization in HeLa cells, but their fluorescence intensities were very low (Supplementary Figure S1). We suspected that the diminished brightness of these indicators might be due to competing interactions between either the CaM or M13 moieties and endogenous protein or peptide-binding partners in the ER, as previously suggested for cameleon-type indicators [29,30]. In an effort to circumvent these problems, we explored the introduction of orthogonal CaM–M13 pairs in R-GECO1 and R-GECO1.2 by mutagenesis of residues involved in interactions at the CaM–M13 interface. An anticipated additional benefit of such changes was a decreased *K*_d_ for binding of Ca^2+^ to the sensor, as reported for cameleon-type indicators with modified CaM–M13 interfaces [12]. To test this idea, mutations were introduced following three different strategies: (1) incorporation of the computationally designed D4 CaM–M13 pair (equivalent to introduction of point mutations V51W, F395A, V411A and L415I, numbered as in the PDB file 3EVR [31]) (Figure 1A) into R-GECO1 [12]; (2) screening of libraries created by randomization of Ala^47^ of R-GECO1.2 (together with point mutation N45I from O-GECO1 [6]), with the reasoning that mutating Ala^47^ could alter the interactions between this residue and the pocket formed by Phe^395^, Met^412^ and Leu^415^ (Figure 1A) from the third EF-hand of CaM; and (3) library creation by randomization of Leu^57^ of M13 in R-GECO1, with the reasoning that mutating Leu^57^ could lead to variants with modified interactions with the hydrophobic pocket formed by Met^354^, Phe^366^ or Met^374^ of CaM (Figure 1A).

Incorporation of the D4 mutations into R-GECO1 (strategy 1) produced a variant, designated low-affinity R-GECO0.1 (LAR-GECO0.1), with a *K*_d_ of ~20 μM and a ~10-fold increase in fluorescence intensity upon binding to Ca^2+^. For the second and third strategies, gene libraries were expressed in *E. coli*, colonies were screened for red fluorescence and promising bright variants were tested for responses to Ca^2+^. From the R-GECO1.2 Ala47X library (strategy 2), we identified a variant, designated LAR-GECO0.2 (R-GECO1.2 with N45I and A47R), with a *K*_d_ of ~8 μM and a ~15-fold increase in fluorescence intensity upon binding to Ca^2+^. From the R-GECO1 Leu57X library (strategy 3), we identified many functional variants, but none with substantially higher *K*_d_ values than R-GECO1. This suggests that modifying Leu^57^ does not significantly alter the affinity for Ca^2+^ or the interactions between CaM and M13.

In an effort to improve the brightness of LAR-GECO0.1 and LAR-GECO0.2 further, we performed three and two rounds, respectively, of directed evolution with selection for variants with bright fluorescence. Specifically, we performed random mutagenesis on LAR-GECO0.1 and LAR-GECO0.2, followed by a screen for colonies with high intensities of red fluorescence. Selected protein variants were extracted from bacterial cultures and tested for both the magnitude of their response to Ca^2+^ as well as their *K*_d_ for binding Ca^2+^. Three to five functional variants with bright red fluorescence and *K*_d_ ~10 μM or higher were then used as templates for the next round of directed evolution. These efforts led to the identification of LAR-GECO1 (derived from LAR-GECO0.1) with seven mutations compared with R-GECO1, a *K*_d_ for Ca^2+^ of 24 μM and a 10-fold increase in fluorescence intensity upon binding to Ca^2+^ (dynamic range), and LAR-GECO1.2 (derived from LAR-GECO0.2) with four mutations compared with R-GECO1.2, a *K*_d_ for Ca^2+^ of 12 μM, and a dynamic range of 8.7 (Figure 1B, Table 1, Supplementary Figure S2 and Supplementary Table S1).

**Table 1:** Properties of new GECOs described in the present paper

Protein	Ca^2+^	*λ*_abs_ (nm) with *ε* (mM^−1^·cm^−1^) in parentheses	*λ*_em_ with *Φ* in parentheses	Brightness* (mM^−1^·cm^−1^)	p*K*_a_	Intensity change‡ ± Ca^2+^	*K*_d_ for Ca^2+^ (μM) with Hill coefficient in parentheses
LAR-GECO1	−	574 (5.3)	598 (0.13)	0.69	8.6	10×	24 (1.3)
	+	561 (35.8)	589 (0.20)	7.2	5.4/8.8†		
LAR-GECO1.2	−	570 (3.6)	594 (0.18)	0.65	9.0	8.7×	12 (1.4)
	+	557 (16.5)	584 (0.34)	5.6	5.8/8.9†		

*Brightness is defined as the product of *ε* and *Φ*.

†In the Ca^2+^-bound state, both LAR-GECO1 and LAR-GECO1.2 show a biphasic effect of pH.

‡Intensity change is measured in 30 mM Mops and 100 mM KCl, pH 7.2, with or without Ca^2+^.

**Figure 2:**
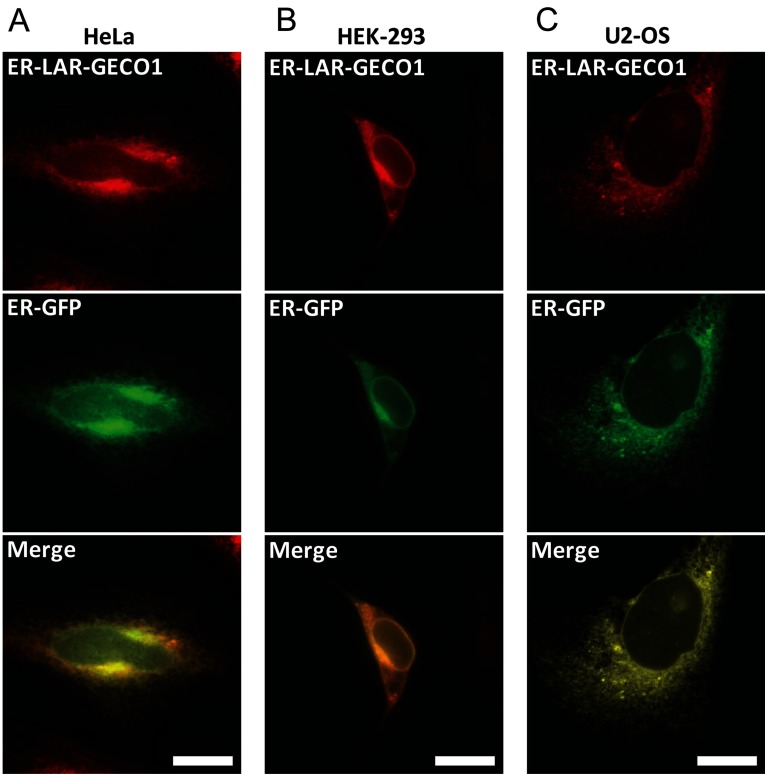
Co-localization of ER-LAR-GECO1 and ER-GFP (**A**) A HeLa cell, (**B**) a HEK-293 cell and (**C**) a U2-OS cell co-expressing ER-LAR-GECO1 and the ER marker ER-GFP [32]. Scale bars=20 μm.

With a *K*_d_ for Ca^2+^ of 24 μM, we reasoned that LAR-GECO1 might be more suitable for detecting the Ca^2+^ dynamics within the ER. To test whether the orthogonal CaM–M13 pair in LAR-GECO1 circumvents the problem of low fluorescence within the ER that we observed for ER-R-GECO1 and ER-R-GECO1.2 (Supplementary Figure S1), an ER-targeted LAR-GECO1 (henceforth termed ER-LAR-GECO1) was generated by fusing it to ER-targeting [23] and ER-retention sequences [24], and was co-expressed with an ER marker (an ER-targeted GFP [32], henceforth termed ER-GFP) in HeLa, HEK-293 and U2-OS cells. Co-expression of ER-LAR-GECO1 and ER-GFP in these three cell lines showed correct ER co-localization (Figure 2). Furthermore, when expressed in HeLa cells, ER-LAR-GECO1 showed significantly higher fluorescence intensity compared with ER-R-GECO1 and ER-R-GECO1.2 (Supplementary Figure S1B), which suggests that the orthogonal CaM–M13 pair in LAR-GECO1 has circumvented its potential interactions with the endogenous proteins.

### *In vitro* characterization of LAR-GECO1 and LAR-GECO1.2

Systematic *in vitro* characterization of LAR-GECO1 and LAR-GECO1.2 revealed that both proteins had spectral properties similar to their progenitors R-GECO1 and R-GECO1.2, respectively (Table 1 and Supplementary Figures S3A–S3D). In addition, LAR-GECO1 and LAR-GECO1.2 exhibit monophasic pH dependencies with apparent p*K*_a_ values of 8.6 and 9.0 respectively in the Ca^2+^-free state. In the Ca^2+^-bound state, both proteins exhibited a biphasic pH dependence with apparent p*K*_a_ values of 5.4/8.8 and 5.8/8.9 respectively (Table 1, and Supplementary Figures S3E and S3F). As a result, both of these Ca^2+^ indicators show large fluorescence changes in response to binding Ca^2+^ at pH values between 6.5 and 8.5, which covers the physiological pH range of the mitochondria and ER.

The decreased Ca^2+^ affinity of LAR-GECO1 (24 μM) compared with its progenitor, R-GECO1 (0.48 μM), is mainly attributed to the D4 mutations (V51W, F395A, V411A and L415I) as they create a new complementary ‘bump-and-hole’ interaction between M13 and CaM [12]. In LAR-GECO1.2, we attribute the 10-fold decrease in Ca^2+^ affinity (from 1.2 μM to 12 μM) to the single mutation, A47R, as the bulky and hydrophilic Arg^47^ probably perturbs the interactions originally present between the side chain of Ala^47^ and the hydrophobic pocket formed by Phe^395^, Met^412^ and Leu^415^ from the third EF-hand of CaM.

### Comparisons of LAR-GECO1 with other low-affinity Ca^2+^ indicators

**Figure 3:**
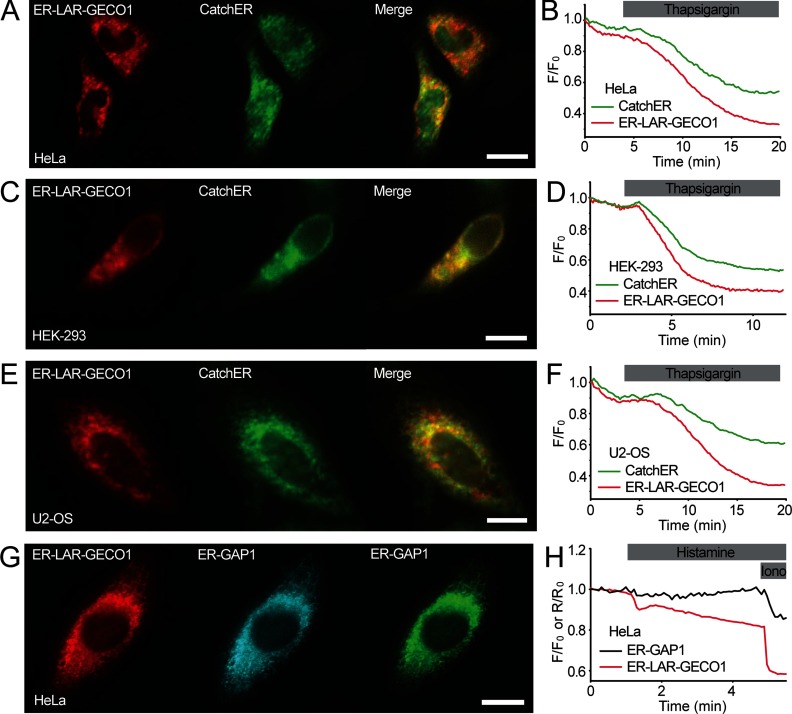
Comparison of ER-LAR-GECO1, CatchER and ER-GAP1 for sensing Ca^2+^ concentration changes within the ER (**A**) HeLa cells, (**C**) a HEK-293 cell and (**E**) a U2-OS cell co-expressing ER-LAR-GECO1 (red) and CatchER (green). Scale bar=20 μm. (**B**, **D** and **F**) Effects of depleting the ER of Ca^2+^ by addition of thapsigargin (10 μM) on the fluorescence intensities of ER-LAR-GECO1 (red line) and CatchER (green line) co-expressed in a HeLa cell, a HEK-293 cell and a U2-OS cell as shown in (**A**), (**C**) and (**E**), respectively. Traces show results from single cells, typical of at least four similar recordings. (**G**) A HeLa cell co-expressing ER-LAR-GECO1 (red) and ER-GAP1 (blue, 395 nm excitation; green, 488 nm excitation). Scale bar=20 μm. (**H**) Effects of the release of ER Ca^2+^ evoked by histamine (100 μM) and ionomycin (Iono, 10 μM) on the fluorescence intensity of ER-LAR-GECO1 (*F*/*F*_0_) and the ratiometric fluorescence response of ER-GAP1 (*R*/*R*_0_) co-expressed in a HeLa cell. Traces are from a single cell, typical of at least four similar recordings.

We compared LAR-GECO1 with the other low-affinity Ca^2+^ indicators, CatchER [20], GAP1 [21] and D1ER [11], in terms of their utility for imaging Ca^2+^ dynamics within the ER of cultured human cells. First, we compared the performance of ER-LAR-GECO1 (red fluorescent) to CatchER (green fluorescent) by two-colour fluorescence imaging of HeLa, HEK-293 and U2-OS cells (Figures 3A–3F) co-transfected with plasmids encoding each of the indicators. Upon treatment with thapsigargin, which inhibits Ca^2+^ uptake into the ER, we observed decreases in the fluorescence intensity of both CatchER and ER-LAR-GECO1 in all three cell lines. The fractional decreases in fluorescence intensity were greater for ER-LAR-GECO1 than for CatchER co-expressed in the same cells (Figures 3B, 3D and 3F). The mean values for the fractional decreases in fluorescence intensity of ER-LAR-GECO1 after addition of thapsigargin to HeLa, U2-OS and HEK-293 cells were 0.46±0.07, 0.56±0.05 and 0.58±0.08, respectively. For CatchER, the values were 0.23±0.06, 0.25±0.05 and 0.50±0.09, respectively (*n*=4–9 cells, from three to four fields; *P*< 0.05 for HeLa and U2-OS cells).

Next, we compared the responses of ER-LAR-GECO1 with those of ER-GAP1 [21] by co-transfecting HeLa cells with plasmids encoding each Ca^2+^ indicator (Figures 3G and 3H). HeLa cells co-expressing both indicators were treated with histamine to stimulate IP_3_-mediated release of Ca^2+^ from intracellular stores, followed by ionomycin to completely deplete intracellular stores of Ca^2+^. In response to histamine, ER-LAR-GECO1 showed fractional decreases in fluorescence intensity (0.08±0.002, *n*=4) that were significantly (*P*< 0.05) greater than the fractional changes in the ratiometric response of ER-GAP1 (0.02±0.007, *n*=4), for which the changes were undetectable in some cells (Figure 3H). The fractional changes evoked by subsequent addition of ionomycin to empty ER Ca^2+^ stores were also significantly larger for ER-LAR-GECO1 (0.27±0.01) than for ER-GAP1 (0.13±0.007; *n*=4; *P*< 0.05) (Figure 3H). This suggests that ER-LAR-GECO1 has greater sensitivity than ER-GAP1 for detecting changes in the concentration of Ca^2+^ within the ER.

**Figure 4:**
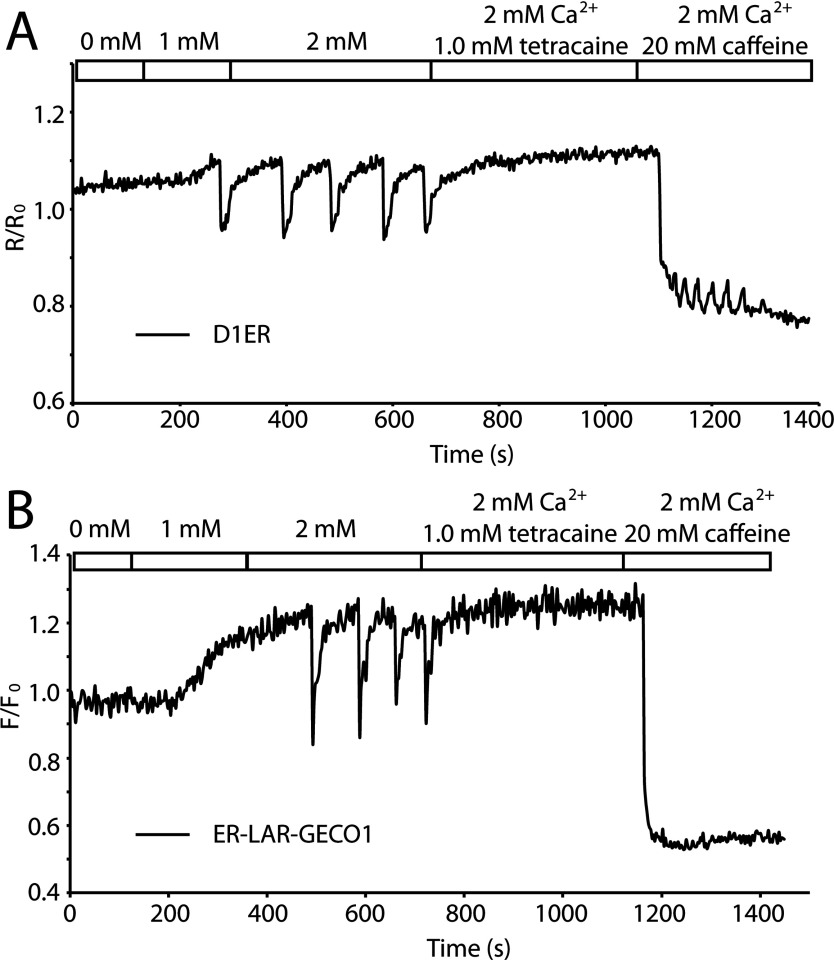
Comparison of ER-LAR-GECO1 and D1ER for measuring SOICR Change of fluorescence ratio (**A**) and intensity (**B**) in HEK-293 cells expressing D1ER (**A**) and ER-LAR-GECO1 (**B**) when the concentration of extracellular Ca^2+^ was changed from 0.0 mM to 2.0 mM to induce SOICR. Caffeine (20 mM) was added to induce release of Ca^2+^ from the ER.

Lastly, we compared ER-LAR-GECO1 with a cameleon-type low-affinity Ca^2+^ indicator, D1ER [11], for measuring store overload-induced Ca^2+^ release (SOICR). HEK-293 cells stably expressing RyR2 were transiently transfected with ER-LAR-GECO1 and D1ER. By increasing the extracellular Ca^2+^ concentration (from 0.0 to 2.0 mM), we observed SOICR as decreases in the YFP/CFP emission ratio of D1ER (Figure 4A) or the fluorescence intensity of ER-LAR-GECO1 (Figure 4B). During SOICR, D1ER showed transient decreases in emission ratio of 37±1% (*n*=64) (Figure 4A) compared with decreases of 39±1% (*n*=72) in the fluorescence intensity of ER-LAR-GECO1 (Figure 4B). Overall, ER-LAR-GECO1 showed comparable performance with that of D1ER in monitoring ER luminal Ca^2+^ in RyR2-expressing HEK-293 cells.

### Imaging mitochondrial Ca^2+^ dynamics in neurons with LAR-GECO1.2

**Figure 5:**
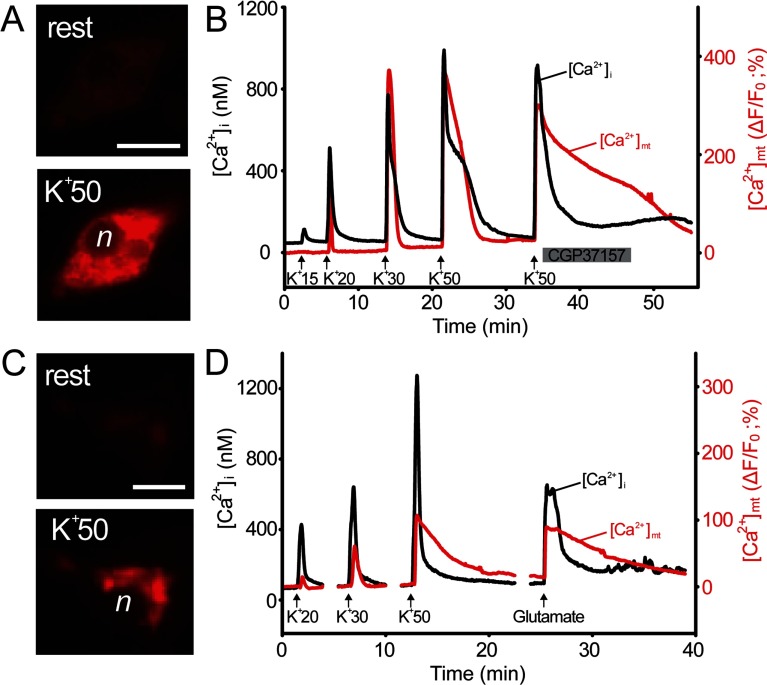
LAR-GECO1.2 for imaging mitochondrial Ca^2+^ in DRG and hippocampal neurons (**A**) Images show mito-LAR-GECO1.2 fluorescence in a DRG neuron from which recordings were made under resting conditions (top) and at the peak of the [Ca^2+^]_mt_ response to 50 mM extracellular K^+^ (bottom; *n*=nucleus), scale bar=20 μm. (**B**) DRG sensory neurons were transfected with a mitochondrially targeted form of LAR-GECO1.2 (mito-LAR-GECO1.2), and subsequently loaded with fura 2 to enable simultaneous measurements of Ca^2+^ concentrations within mitochondria ([Ca^2+^]_mt_; red trace) and cytosol ([Ca^2+^]_i_; black trace). DRG neurons were depolarized using 15 mM, 20 mM, 30 mM and 50 mM KCl (30 s) in extracellular solution (vertical arrows). The inhibitor of mitochondrial Na^+^/Ca^2+^ exchange, CGP37157 (10 μM), reversibly inhibited extrusion of Ca^2+^ from mitochondria (red) and eliminated the [Ca^2+^]_i_ plateau that immediately followed the peak rise in [Ca^2+^]_i_ in control cells. (**C**) Images show mito-LAR-GECO1.2 fluorescence in a hippocampal neuron from which recordings were made in the resting state (top) and at the peak elevation of [Ca^2+^]_mt_ induced by 50 mM extracellular K^+^ (bottom; *n*=nucleus), scale bar=10 μm. (**D**) Simultaneous imaging of cytosolic ([Ca^2+^]_i_, black) and mitochondrial ([Ca^2+^]_mt_, red) concentrations of Ca^2+^ in mouse hippocampal neurons using fura 2 and mito-LAR-GECO1.2, respectively. Elevation of [Ca^2+^]_i_ and [Ca^2+^]_mt_ were induced by 30-s depolarizations evoked by KCl (20 mM, 30 mM or 50 mM) or 100 μM glutamate (+10 μM glycine); 200 nM tetrodotoxin was present throughout the recordings to block action potentials.

In neurons, mitochondria buffer Ca^2+^ influx during excitation, taking up Ca^2+^ via the Ca^2+^ uniporter and then releasing Ca^2+^ back into the cytosol via Na^+^/Ca^2+^ and H^+^/Ca^2+^ exchangers. This buffering results in transient elevations of [Ca^2+^]_mt_ during neuronal activity [33,34]. These mitochondrial Ca^2+^ signals play important roles in ATP synthesis, neuronal plasticity and survival [35–37]. To determine whether LAR-GECO1.2 could be used to image these activity-dependent mitochondrial Ca^2+^ elevations in neurons, we imaged cultured peripheral and central neurons using a mitochondrially targeted form of the indicator, mito-LAR-GECO1.2 (Figure 5). DRG sensory neurons were depolarized by application of high concentrations of K^+^ (15 mM, 20 mM, 30 mM or 50 mM extracellular KCl for 30 s), and the fluorescence of mito-LAR-GECO1.2 and fura-2 were used to simultaneously measure [Ca^2+^]_mt_ and the cytosolic Ca^2+^ concentration [Ca^2+^]_i_, respectively. The results showed that depolarization evoked coincident and rapidly reversible increases in [Ca^2+^]_mt_ and [Ca^2+^]_i_ (Figure 5B). Treatment with an inhibitor of the mitochondrial Na^+^/Ca^2+^ exchanger, CGP37157 (10 μM), slowed the recovery of the increase in [Ca^2+^]_mt_, and this effect was reversible (Figure 5B). In hippocampal neurons, KCl-evoked depolarization or application of glutamate also produced elevations of [Ca^2+^]_i_ and [Ca^2+^]_mt_, although the amplitudes of the increases in [Ca^2+^]_mt_ were smaller than those produced by similar stimuli in DRG neurons (Figure 5D). These observations using mito-LAR-GECO1.2 are consistent with the known properties of mitochondrial Ca^2+^ transport in neurons [33,36,38].

### Dual-colour imaging of cytosolic and ER luminal Ca^2+^ using ER-LAR-GECO1

**Figure 6:**
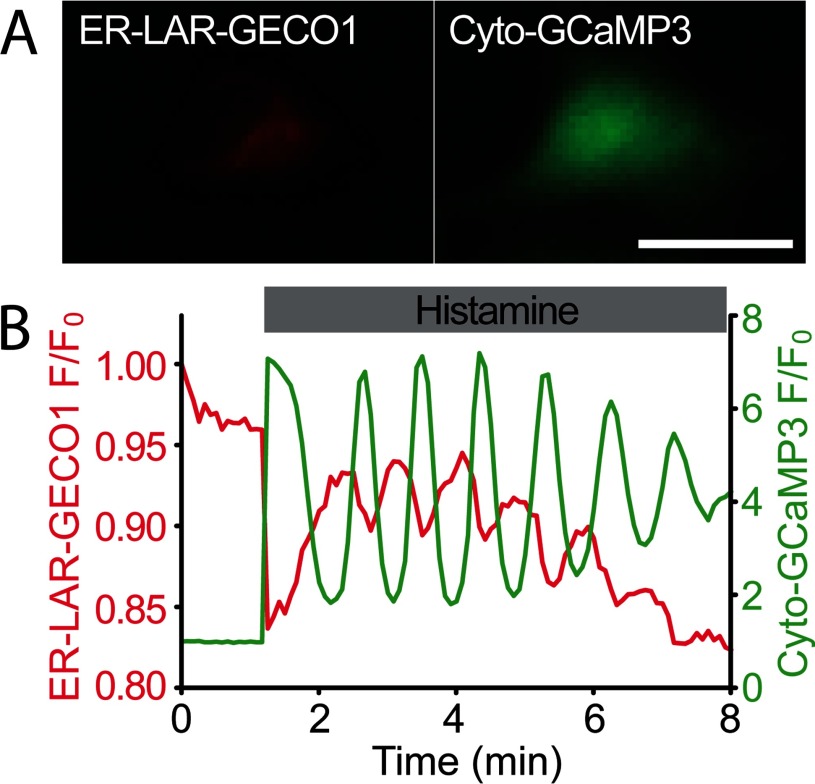
Dual-colour imaging of cytosolic and ER luminal Ca^2+^ using ER-LAR-GECO1 and Cyto-GCaMP3 (**A**) A HeLa cell co-expressing ER-LAR-GECO1 (left panel) and Cyto-GCaMP3 (right panel). Images correspond to the time of maximal increase in the fluorescence intensity of Cyto-GCaMP3 after treatment with histamine (100 μM). Scale bar=20 μm. (**B**) Changes of the fluorescence intensities of ER-LAR-GECO1 (red trace) and Cyto-GCaMP3 (green trace) in a HeLa cell upon treatment with histamine (100 μM). This trace is representative of at least five similar recordings.

To explore the possibility of using LAR-GECO1 and a green fluorescent indicator for dual-colour imaging of Ca^2+^ dynamics within the cytosol and ER lumen, we co-expressed a green cytosolic Ca^2+^ indicator, GCaMP3 [39] (henceforth termed Cyto-GCaMP3), and ER-LAR-GECO1 in cells. Upon stimulation with histamine, we observed oscillatory increases in the fluorescence of Cyto-GCaMP3 that were coincident with oscillatory decreases in the fluorescence of ER-LAR-GECO1 (Figure 6). These results demonstrate that the indicators can report oscillatory release of Ca^2+^ from the ER into the cytosol. We also co-expressed ER-LAR-GECO1 with another cytosolic green Ca^2+^ indicator, G-GECO1.1 [5] (henceforth termed Cyto-G-GECO1.1) in HeLa, HEK-293 and U2-OS cells respectively. In all three cell lines, ER-LAR-GECO1 reported changes in the concentration of Ca^2+^ within the ER that showed close temporal coincidence with opposing changes in the cytosolic Ca^2+^ signals reported by Cyto-G-GECO1.1 (Supplementary Figure S4). In most cells, the fluorescence intensity of ER-LAR-GECO1 declined transiently and then recovered, mirroring the time course of the cytosolic Ca^2+^ signals reported by Cyto-G-GECO1.1 (Supplementary Figures S4D–S4F). The recovery of the fluorescence intensity of ER-LAR-GECO1 was observed in nominally Ca^2+^-free medium (Supplementary Figures S4G–S4I) and it was inhibited by thapsigargin (Supplementary Figures S4J–S4L). These observations are consistent with re-uptake of Ca^2+^ from the cytosol occurring via the sarcoplasmic/endoplasmic reticulum Ca^2+^-ATPase (SERCA).

### Conclusion

By utilizing rational design and directed evolution, we have developed two low-affinity red fluorescent Ca^2+^ indicators, LAR-GECO1 and LAR-GECO1.2, with *K*_d_ values for binding to Ca^2+^ of 24 μM and 12 μM respectively. These indicators enable robust detection of Ca^2+^ dynamics in organelles containing relatively high Ca^2+^ concentrations, as we demonstrate for the mitochondria in primary neurons and the ER in cultured human cells. Furthermore, comparisons of LAR-GECO1 with other FP-based low-affinity Ca^2+^ indicators demonstrate that LAR-GECO1 is a sensitive single-wavelength Ca^2+^ indicator for detecting Ca^2+^ dynamics within the ER of mammalian cells. In addition, LAR-GECO1 enables simultaneous dual-colour imaging with GFP-based fluorescent probes.

## Online data

Supplementary data
