# Supplementary material for: Red fluorescent genetically encoded Ca2+ indicators for use in mitochondria and endoplasmic reticulum
Source: Biochem J. 2014 Oct 23;464(Pt 1):13–22. doi: 10.1042/BJ20140931 (PMC4214425; doi:10.1042/BJ20140931)
Supplement: Supplementary data [file bj4640013ntsadd.pdf]

SUPPLEMENTARY ONLINE DATA

Red fluorescent genetically encoded Ca^{2+} indicators for use in mitochondria and endoplasmic reticulum

Jiahui WU[*], David L. PROLE[†], Yi SHEN[*], Zhihong LIN[‡], Aswini GNANASEKARAN[‡], Yingjie LIU[§], Lidong CHEN[*], Hang ZHOU[*], S.R. Wayne CHEN[§‖], Yuriy M. USACHEV[‡], Colin W. TAYLOR[†] and Robert E. CAMPBELL[*1]

[*]Department of Chemistry, University of Alberta, Edmonton, Alberta T6G 2G2, Canada
[†]Department of Pharmacology, University of Cambridge, Cambridge CB2 1PD, UK
[‡]Department of Pharmacology, University of Iowa Carver College of Medicine, Iowa City, IA 52242, USA
[§]Department of Physiology and Pharmacology, Libin Cardiovascular Institute of Alberta, University of Calgary, Calgary, Alberta, T2N 4N1, Canada
‖Department of Biochemistry and Molecular Biology, Libin Cardiovascular Institute of Alberta, University of Calgary, Calgary, Alberta, T2N 4N1, Canada

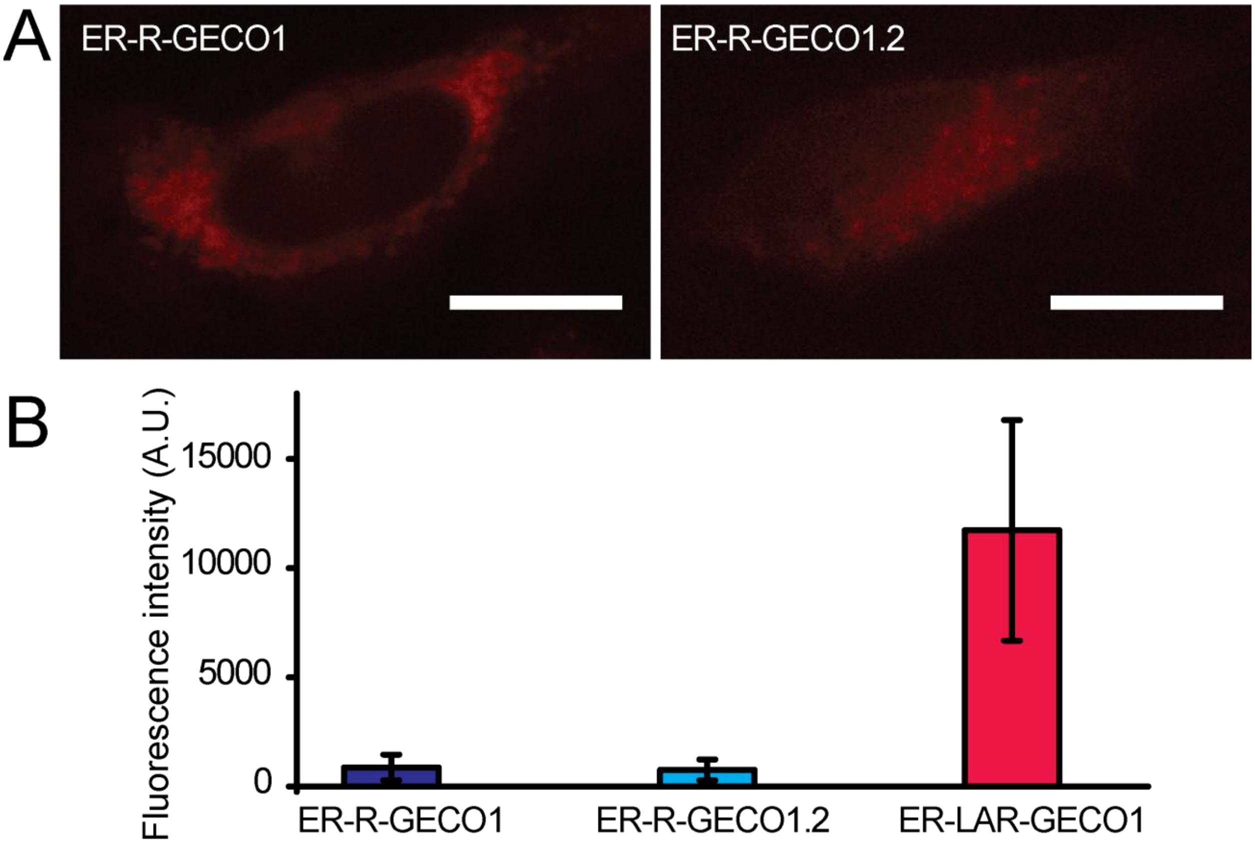

Figure S1 Diminished brightness of R-GECO1 and R-GECO1.2 in the ER of HeLa cells

(A) HeLa cells expressing ER-R-GECO1 (left panel) and ER-R-GECO1.2 (right panel), scale bar = 20 μm.
(B) Comparison of the red fluorescence intensities of ER-R-GECO1 (n = 67 cells; $p << 0.001$), ER-R-GECO1.2 (86 cells; $p << 0.001$) and ER-LAR-GECO1 (57 cells) in HeLa cells.

GCaMP #s	39	40	41	42	43	44	45	46	47	48	49	50	51	52	53	54	55	56	57	58	59	60	61	62	63	64	65	66	67	68	69	70	71	72	73	74	75	76	77	78
R-GECO1	S	S	R	R	K	W	N	K	A	G	H	A	V	R	A	I	G	R	L	S	S	P	V	-	V	S	E	R	M	Y	P	E	D	G	A	L	K	S	E	I
LAR-GECO1	S	S	R	R	K	W	N	K	A	G	H	A	**W**	R	A	I	G	R	L	S	S	P	V	-	V	S	E	R	M	Y	P	E	D	G	A	L	K	S	E	I
R-GECO1.2	S	S	R	R	K	W	N	K	A	G	H	A	V	R	A	I	G	R	L	S	S	P	V	-	V	S	E	R	M	Y	P	E	D	G	A	L	K	S	E	I
LAR-GECO1.2	S	S	R	R	K	W	**I**	K	**R**	G	H	A	V	R	A	I	G	R	L	S	S	P	V	-	V	S	E	R	M	Y	P	E	D	G	A	L	K	S	E	I

GCaMP #s	79	80	81	82	83	84	85	86	87	88	89	90	91	92	93	94	95	96	97	98	99	100	101	102	103	104	105	106	107	108	109	110	111	112	113	114	115	116	117	118
R-GECO1	K	K	G	L	R	L	K	D	G	G	H	Y	A	A	-	-	E	V	K	T	T	Y	K	A	K	K	P	V	Q	L	P	G	A	Y	I	V	D	I	K	L
LAR-GECO1	K	K	G	L	R	L	K	D	G	G	H	Y	A	A	-	-	E	V	K	T	T	Y	K	A	K	K	P	V	Q	L	P	G	A	Y	**V**	V	D	I	K	L
R-GECO1.2	K	K	G	L	R	L	K	D	G	G	H	Y	A	A	-	-	E	V	K	T	T	Y	K	A	K	K	P	V	Q	L	P	G	A	Y	I	V	D	I	K	L
LAR-GECO1.2	K	K	G	L	R	L	K	D	G	G	H	Y	A	A	-	-	E	V	K	T	T	Y	K	A	K	K	P	V	Q	L	P	G	A	Y	I	V	D	I	K	L

GCaMP #s	119	120	121	122	123	124	125	126	127	128	129	130	131	132	133	134	135	136	137	138	139	140	141	142	143	144	145	146	147	148	149	150	151	152	153	154	155	156	157	158
R-GECO1	D	I	V	S	-	H	N	E	D	Y	T	I	V	E	Q	C	E	R	A	E	G	R	H	S	T	G	G	M	D	E	L	Y	K	G	G	T	G	G	S	L
LAR-GECO1	D	I	V	S	-	H	N	E	D	Y	T	I	V	E	Q	C	E	R	A	E	G	R	H	S	T	G	G	M	D	E	L	Y	K	G	G	T	G	G	S	L
R-GECO1.2	D	I	V	S	-	H	N	E	D	Y	T	I	V	E	Q	C	E	R	A	E	G	R	H	S	T	G	G	M	D	E	L	Y	K	G	G	T	G	G	S	L
LAR-GECO1.2	D	I	V	S	-	H	N	E	D	Y	T	I	V	E	Q	C	E	R	A	**V**	G	R	H	S	T	G	G	M	D	E	L	Y	K	G	G	T	G	G	S	L

GCaMP #s	159	160	161	162	163	164	164a	164b	164c	164d	165	166	167	168	169	170	171	172	173	174	175	176	177	178	179	180	181	182	183	184	185	186	187	188	189	190	191	192	193	194
R-GECO1	V	S	K	G	E	E	D	N	M	A	I	I	K	E	F	M	R	F	K	V	H	M	E	G	S	V	N	G	H	E	F	E	I	E	G	E	G	E	G	R
LAR-GECO1	V	S	K	G	E	E	D	N	M	A	I	I	K	E	F	M	R	F	K	V	H	M	E	G	S	V	N	G	H	E	F	E	I	E	G	E	G	E	G	R
R-GECO1.2	V	S	K	G	E	E	D	N	R	A	I	V	K	E	F	M	R	F	K	L	H	M	E	G	S	V	N	G	H	E	F	E	I	E	G	E	G	E	G	R
LAR-GECO1.2	V	S	K	G	E	E	D	N	R	A	I	V	K	E	F	M	R	F	K	L	H	M	E	G	S	V	N	G	H	E	F	E	I	E	G	E	G	E	G	R

GCaMP #s	195	196	197	198	199	200	201	202	203	204	205	206	207	208	209	209a	210	211	212	213	214	215	216	217	218	219	220	221	222	223	224	225	226	227	228	229	230	231	232	233
R-GECO1	P	Y	E	A	F	Q	T	A	K	L	K	V	T	K	G	G	P	L	P	F	A	W	D	I	L	S	P	Q	F	M	Y	G	S	K	A	Y	I	K	H	P
LAR-GECO1	P	Y	E	A	F	Q	T	A	K	L	K	V	T	K	G	G	P	L	P	F	A	W	D	I	L	S	P	Q	F	M	Y	G	S	K	A	Y	I	K	H	P
R-GECO1.2	P	Y	E	A	F	Q	T	A	K	L	K	V	T	K	G	G	P	L	P	F	A	W	D	I	L	S	P	Q	L	M	Y	G	S	K	A	Y	I	K	H	P
LAR-GECO1.2	P	Y	E	A	F	Q	T	A	K	L	K	V	T	K	G	G	P	L	P	F	A	W	D	I	L	S	P	Q	L	M	Y	G	S	K	A	Y	I	K	H	P

GCaMP #s	234	235	236	237	238	239	240	241	242	243	244	245	246	247	248	249	250	251	252	253	254	255	256	257	258	259	260	261	262	263	264	265	266	267	268	269	270	271	272	273
R-GECO1	A	D	I	P	-	-	D	Y	F	K	L	S	F	P	E	G	F	R	W	E	R	V	M	N	F	E	D	G	G	I	I	H	V	N	Q	D	S	S	L	Q
LAR-GECO1	A	D	I	P	-	-	D	Y	F	K	L	S	F	P	E	G	F	R	W	E	R	V	M	N	F	E	D	G	G	I	I	H	V	N	Q	D	S	S	L	Q
R-GECO1.2	A	D	I	P	-	-	D	Y	F	K	L	S	F	P	E	G	F	R	W	E	R	V	M	N	F	E	D	G	G	I	I	H	V	S	Q	D	T	S	L	Q
LAR-GECO1.2	A	D	I	P	-	-	D	Y	F	K	L	S	F	P	E	G	F	R	W	E	R	V	M	N	F	E	D	G	G	I	I	H	V	S	Q	D	T	S	L	Q

GCaMP #s	274	275	276	277	278	279	280	281	282	283	284	285	286	287	288	289	290	291	292	293	294	295	296	297	298	299	300	300a	300b	301	302	303	304	305	306	307	308	309	310	311
R-GECO1	D	G	V	F	I	Y	K	V	K	L	R	G	T	N	F	P	P	D	G	P	V	M	Q	K	K	T	M	G	W	E	A	T	R	D	Q	L	T	E	E	Q
LAR-GECO1	D	G	V	F	I	Y	K	V	K	L	R	G	T	N	F	P	P	D	G	P	V	M	Q	K	K	T	M	G	W	E	A	T	R	D	Q	L	T	E	E	Q
R-GECO1.2	D	G	V	F	I	Y	K	V	K	L	R	G	T	N	F	P	P	D	G	P	V	M	Q	K	K	T	M	G	W	E	A	T	R	D	Q	L	T	E	E	Q
LAR-GECO1.2	D	G	V	F	I	Y	K	V	K	L	R	G	T	N	F	P	P	D	G	P	V	M	Q	K	K	T	M	G	W	E	A	T	R	D	Q	L	T	E	E	Q

GCaMP #s	312	313	314	315	316	317	318	319	320	321	322	323	324	325	326	327	328	329	330	331	332	333	334	335	336	337	338	339	340	341	342	343	344	345	346	347	348	349	350	351
R-GECO1	I	A	E	F	K	E	A	F	S	L	F	D	K	D	G	D	G	T	I	T	T	K	E	L	G	T	V	M	R	S	L	G	Q	N	P	T	E	A	E	L
LAR-GECO1	I	A	E	F	K	E	A	F	S	L	F	D	K	D	G	D	G	T	I	T	T	K	E	L	G	T	V	M	R	S	L	G	Q	N	P	T	E	A	E	L
R-GECO1.2	I	A	E	F	K	E	A	F	S	L	F	D	K	D	G	D	G	T	M	T	T	K	E	L	G	T	V	M	R	S	L	G	Q	N	P	T	E	A	E	L
LAR-GECO1.2	I	A	E	F	K	E	A	F	S	L	F	D	**E**	D	G	D	G	T	M	T	T	K	E	L	G	T	V	M	R	S	L	G	Q	N	P	T	E	A	E	L

GCaMP #s	352	353	354	355	356	357	358	359	360	361	362	363	364	365	366	367	368	369	370	371	372	373	374	375	376	377	378	379	380	381	382	383	384	385	386	387	388	389	390	391
R-GECO1	Q	D	M	I	N	E	V	D	A	D	G	D	G	T	F	D	F	P	E	F	L	T	M	M	A	R	K	M	N	D	T	D	S	E	E	E	I	R	E	A
LAR-GECO1	Q	D	M	I	**S**	E	V	D	A	D	G	D	G	T	F	D	F	P	E	F	L	T	M	M	A	R	K	M	N	**Y**	T	D	S	E	E	E	I	R	E	A
R-GECO1.2	Q	D	M	I	N	E	V	D	A	D	G	D	G	T	F	D	F	P	E	F	L	T	M	M	A	R	K	M	N	D	T	D	S	E	E	E	I	R	E	A
LAR-GECO1.2	Q	D	M	I	N	E	V	D	A	D	G	D	G	T	F	D	F	P	E	F	L	T	M	M	A	R	K	M	N	D	T	D	S	E	E	E	I	R	E	A

GCaMP #s	392	393	394	395	396	397	398	399	400	401	402	403	404	405	406	407	408	409	410	411	412	413	414	415	416	417	418	419	420	421	422	423	424	425	426	427	428	429	430	431
R-GECO1	F	R	V	F	D	K	D	G	N	G	Y	I	G	A	A	E	L	R	H	V	M	T	D	L	G	E	K	L	T	D	E	E	V	D	E	M	I	R	V	A
LAR-GECO1	F	R	V	**A**	D	K	D	G	N	G	Y	I	G	A	A	E	L	R	H	**A**	M	T	D	**I**	G	E	K	L	T	D	E	E	V	D	E	M	I	R	V	A
R-GECO1.2	F	R	V	F	D	K	D	G	N	G	Y	I	G	A	A	E	L	R	H	V	M	T	D	L	G	E	K	I	T	D	E	E	V	D	E	M	I	R	V	A
LAR-GECO1.2	F	R	V	F	D	K	D	G	N	G	Y	I	G	A	A	E	L	R	H	V	M	T	D	L	G	E	K	I	T	D	E	E	V	D	E	M	I	R	V	A

GCaMP #s	432	433	434	435	436	437	438	439	440	441	442	443	444	445	446	447	448	449	450	451
R-GECO1	D	I	D	G	D	G	Q	V	N	Y	E	E	F	V	Q	M	M	T	A	K
LAR-GECO1	D	I	D	G	D	G	Q	V	N	Y	E	E	F	V	Q	M	M	T	A	K
R-GECO1.2	D	I	D	G	D	G	Q	V	N	Y	E	E	F	V	Q	M	M	T	A	K
LAR-GECO1.2	D	I	D	G	D	G	Q	V	N	Y	E	E	F	V	Q	M	M	T	A	K

Figure S2 Sequence Alignment of R-GECO1, LAR-GECO1, R-GECO1.2 and LAR-GECO1.2

Changes in LAR-GECO1 relative to R-GECO1 are shown as green boxes and in LAR-GECO1.2 relative to R-GECO1.2 are shown as pink boxes. Residue numbering is consistent with the crystal structure of GCaMP2 (PDB ID 3EVR) [31].

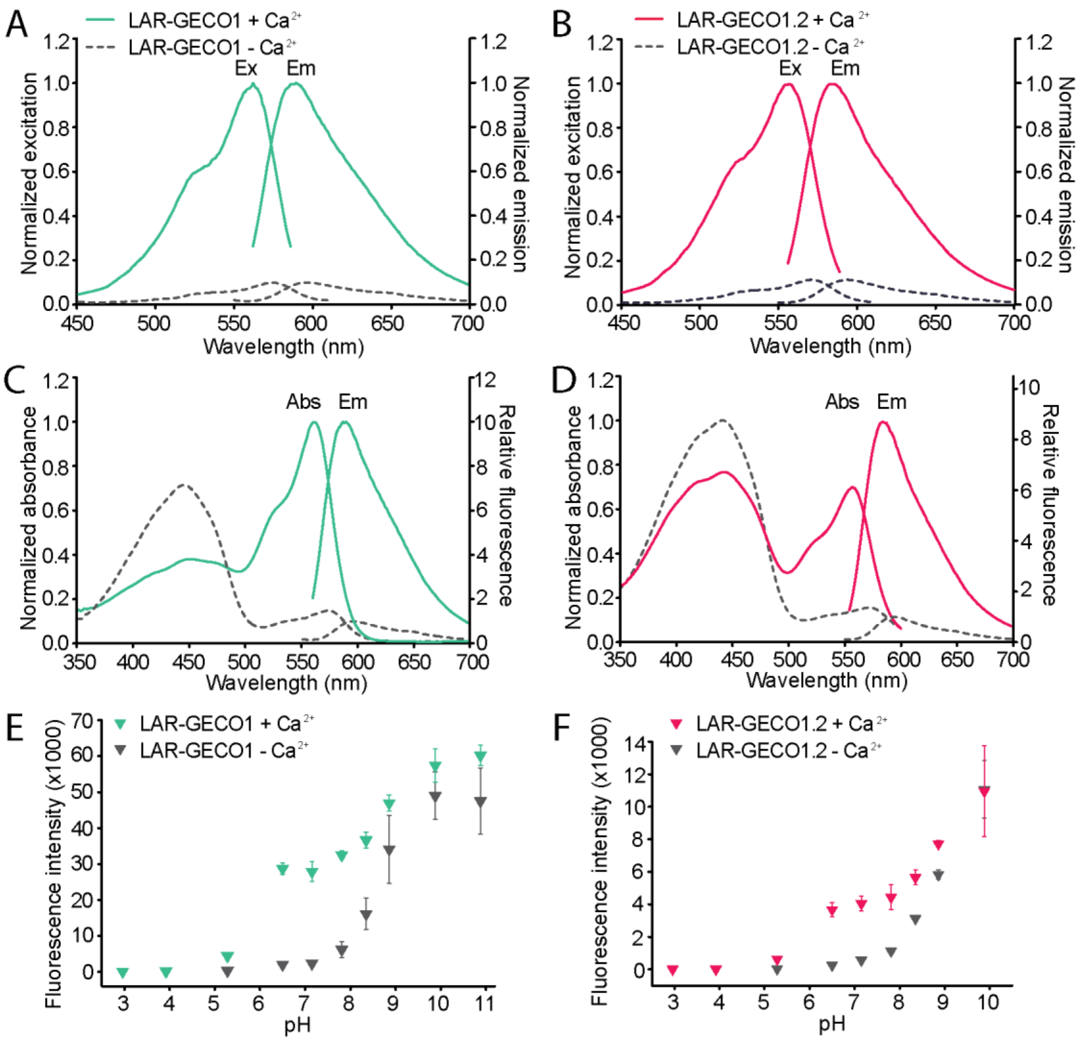

Figure S3 Characterization of LAR-GECO1 and LAR-GECO1.2

For (A-D), the Ca^{2+}-free state is represented with a dotted line and the Ca^{2+}-bound state by a solid line. All spectra were measured as previously described [6].

(A and B) Excitation (Ex) and emission (Em) spectra of LAR-GECO1 (A) and LAR-GECO1.2 (B) in the Ca^{2+}-free and Ca^{2+}-bound states.

(C and D) Absorbance (Abs) and emission (Em) spectra of LAR-GECO1 (C) and LAR-GECO1.2 (D) in the Ca^{2+}-free and Ca^{2+}-bound states.

(E and F) Fluorescence intensities of LAR-GECO1 (E) and LAR-GECO1.2 (F) as a function of pH.

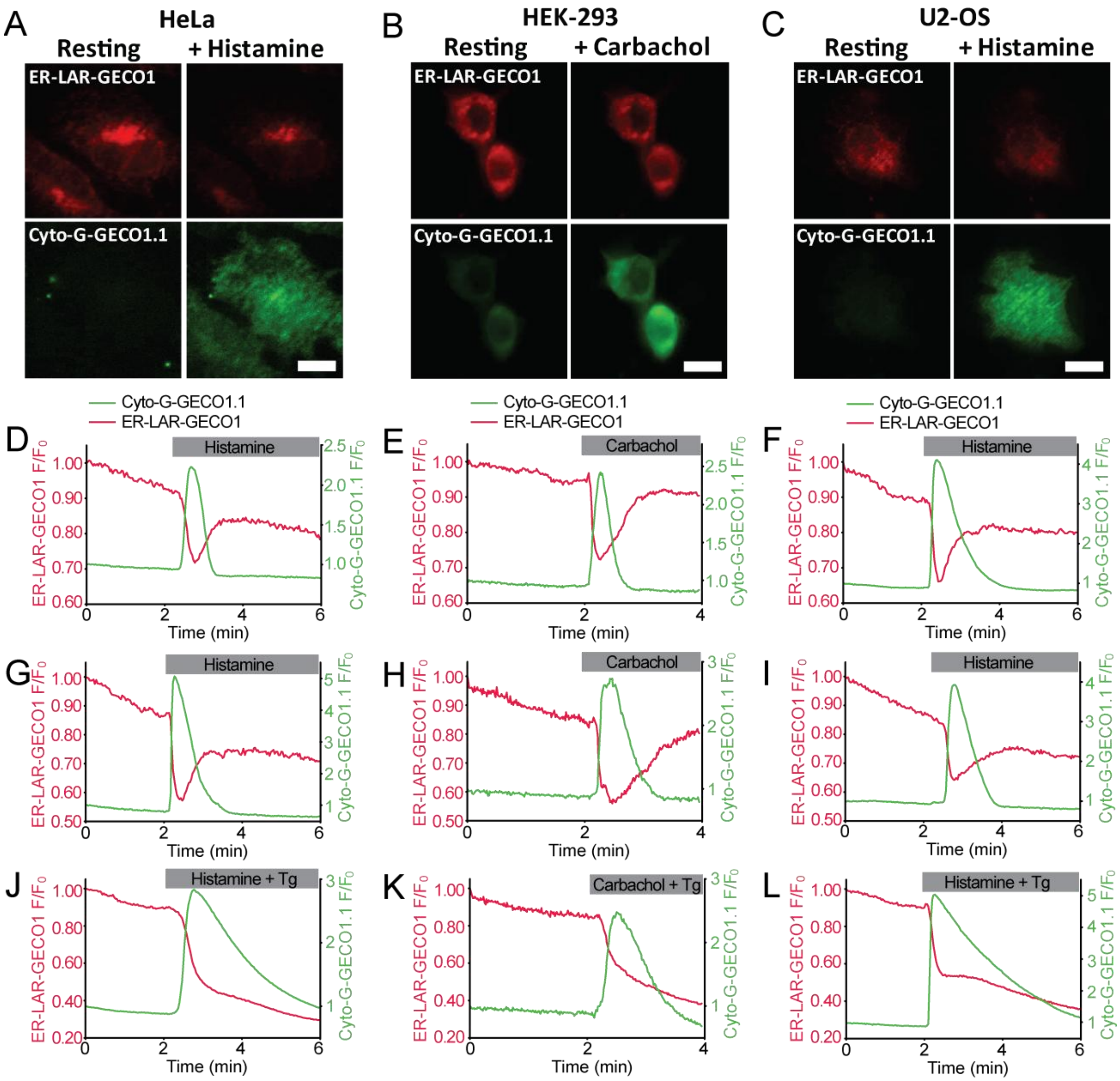

Figure S4 Characterization of ER-LAR-GECO1 in HeLa, HEK-293 and U2-OS cells

(A) HeLa cells, (B) HEK-293 cells and (C) a U2-OS cell co-expressing ER-LAR-GECO1 (red) and Cyto-G-GECO1.1 (green), imaged in the resting state (left panels) and after treatment with histamine (100 µM, right panels). Images after treatment with histamine correspond to the time of maximal increase in the fluorescence intensity of Cyto-G-GECO1.1. Scale bars = 10 µm.
(D-L) Effects of the release of ER Ca^{2+} evoked by histamine (100 µM) or carbachol (1 mM) applied alone or with thapsigargin (Tg, 10 µM), on the fluorescence intensities of ER-LAR-GECO1 (red lines) and Cyto-G-GECO1.1 (green lines) co-expressed in HeLa cells (DGJ), HEK-293 cells (EHK) and U2-OS cells (FIL). Cells were bathed in HBS (D-F and J-L) or nominally Ca^{2+}-free HBS (G-I). Each trace shows the response from a single cell, and is representative of at least 4 similar recordings.

Table S1 List of substitutions for new GECOs described in this work. Residues are numbered as described in **Figure S2**

Protein	LAR-GECO1 substitutions relative to R-GECO1<br>LAR-GECO1.2 substitutions relative to R-GECO1.2
LAR-GECO1	V51W, I113V, N356S, D381Y, F395A, V411A, L415I
LAR-GECO1.2	N45I, A47R, E138V, K324E
